# Dietary quality of predominantly traditional diets is associated with blood glucose profiles, but not with total fecal *Bifidobacterium* in Indonesian women

**DOI:** 10.1371/journal.pone.0208815

**Published:** 2018-12-21

**Authors:** Shiela Stefani, Sanny Ngatidjan, Monica Paotiana, Kurnia A. Sitompul, Murdani Abdullah, Dyah P. Sulistianingsih, Anuraj H. Shankar, Rina Agustina

**Affiliations:** 1 Department of Nutrition, Faculty of Medicine, Universitas Indonesia—Dr. Cipto Mangunkusumo General Hospital, Jakarta, Indonesia; 2 Department of Internal Medicine, Faculty of Medicine Universitas Indonesia—Dr. Cipto Mangunkusumo General Hospital, Jakarta, Indonesia; 3 Human Nutrition Research Center, Indonesian Medical Education and Research Institute, Faculty of Medicine, Universitas Indonesia, Jakarta, Indonesia; 4 Department of Nutrition, Harvard T.H. Chan School of Public Health, Harvard University, Boston, MA, United States of America; 5 Southeast Asian Ministers of Education Organization Regional Centre for Food and Nutrition (SEAMEO RECFON)/ Pusat Kajian Gizi Regional (PKGR), Universitas Indonesia, Jakarta, Indonesia; University of Adelaide School of Medicine, AUSTRALIA

## Abstract

**Background:**

A high quality modern diet is associated with reduced risk of metabolic disease and diabetes. However, it remains unclear whether the quality of predominantly traditional ethnic diets is associated with such conditions. Moreover, the relationship between dietary quality and microbiota, a potential mediator of metabolic disease, has not been studied.

**Objective:**

We investigated the relationship of dietary quality of traditional ethnic diets in Indonesia with fasting blood glucose (FBG), HbA1c, and the number of fecal *Bifidobacterium*.

**Design:**

A cross-sectional study was conducted in selected districts with predominantly animal- or plant-based traditional diets of West Sumatera and West Java provinces, respectively. A total of 240 apparently healthy women aged 19–50 years were randomly selected from 360 women screened by a cluster sampling design. Dietary quality was assessed by 2-day repeated 24-hour food recall, and scored using the Healthy Eating Index (HEI) 2010. FBG was quantified with the enzymatic colorimetric method, and HbA1c by using hexokinase and high-performance liquid chromatography, and total fecal *Bifidobacterium* by real-time quantitative polymerase chain reaction.

**Results:**

The HEI scores of 99% of women were <51, indicating a low-quality diet. In adjusted multivariate regression, HEI was inversely associated with FBG (*ß* = -0.403; 95% CI = -0.789 to -0.016; p = 0.041) and HbA1c (*ß* = -0.018; 95% CI = -0.036 to 0.000; p = 0.048) but was not significantly associated with total levels of *Bifidobacterium* (*ß* = -0.007, p = 0.275). *Bifidobacterium* count was not significantly associated with either FBG or HbA1c levels.

**Conclusion:**

Low dietary quality is clearly associated with risk of increased markers of blood glucose. However, any mediating role of *Bifidobacterium* between dietary quality and glucose outcomes was not apparent. Innovative interventions for healthy eating should be implemented to increase dietary quality of populations transitioning from predominantly traditional to modern diets, to reduce the risk of diabetes, especially in women.

## Introduction

Type 2 diabetes is a global public health crisis and is rapidly increasing in low- and middle-income countries (LMIC) [1]. Indonesia is one of the top ten countries in the number of diabetes patients [2], and diabetes accounts for 6.5% of total deaths nationally [3], with mortality risk in women twice as high as for men [4]. Rapid urbanization, increasingly sedentary lifestyles, hormonal influences, and low-quality diets, especially for women, are major factors affecting the risk of diabetes, and associated metabolic disease [5].

The gut microbiota, the largest symbiont community of the human organism, emerges as a pivotal player in the relationship between diet and health [6]. Several factors may influence the changes in composition of the gastrointestinal microbiota, including age, genetics, immunity, and diets [7]. Recent evidence suggests that modification of gut bacterial populations can improve health [8], and modulate the link between diet and metabolic disease [9]. In this regard, *Bifidobacterium* warrant attention.

The *Bifidobacterium* genus currently comprises 48 recognized species. *Bifidobacterium* are commensal gram-positive bacteria, and several genome sequences define specific macromolecules associated with host-microbiome interactions and with important roles in maintaining the intestinal epithelial integrity and permeability, and production of anti-inflammatory metabolites [10–14]. Diets high in saturated fatty acids [8, 15] and low in fiber [16] are associated with reduced total number of *Bifidobacterium*, and are linked to overweight and obesity [17], and elevated levels of lipopolysaccharide (LPS) in the circulation. These conditions can lead to low-grade chronic inflammation [18], which hastens progression of non-communicable disease [19], including diabetes mellitus. Oral consumption of certain strains has been linked to specific health effects. For example, *Bifidobacterium infantis* 35624 may reduce systemic pro inflammatory biomarkers [20]; *Bifidobacterium lactis* HN019 can improve gut transit time and decrease signs of functional gastrointestinal disorders in adults [21]; *Bifidobacterium pseudocatenulatum* CECT 7765 together with dietary recommendations can decrease the high-sensitivity C-reactive protein marker and monocyte chemoattractant protein-1, and increase high-density lipoprotein cholesterol and omentin-1 [22]; and a mixture of *Bifidobacterium lactis* Bi1, *B*. *breve* Bbr8 and *B*. *breve* BL10 was able to both prevent and ameliorate established obesity by reducing weight gain, adipose tissue fat accumulation, adipocyte size, and macrophage and CD4^+^ T cell infiltration, and improving lipid profiles and regulate leptin and cytokine secretion.[23]

Effects of microbiota on metabolism may be further modulated in women as pregnancy can change intestinal microbiota composition,[24] and progesterone and estrogen can also affect glucose metabolism [25]. Understanding the role of diet as a primary contributor to changes in gut microbiota and metabolic profile, and providing food-based recommendations that are acceptable and effective is critical to design of interventions to modulate gut microbiota and improve metabolic outcomes. Therefore, studies focused on dietary quality, blood glucose and *Bifidobacterium* status in active reproductive aged women are needed.

The Ministry of Health of the Republic of Indonesia has issued Indonesian dietary guidelines, but implementation remains poor, resulting in high prevalence of overweight and obesity, particularly in adult women [26]. Indeed, providing such recommendations is challenging due to the high diversity of dietary patterns within modern diets and across ethnicities in LMICs, including Indonesia with more than 700 ethnic groups. More tailored guidelines that accommodate healthy ethnic dietary practices are therefore needed. Detailed study of such diets and their associations with health status are required.

The Minangkabau ethnicity in West Sumatera and Sundanese in West Java provinces have well known traditional foods that are widely consumed in Indonesia, and present a paradox in dietary habits. The Minangkabau prefer animal-based foods (beef, lamb, chicken, fish) as compared to vegetables [27], while the Sundanese consume more plant-based foods such as vegetables and fruits [28]. Unfortunately, examination of the health effects of these diets has been hampered by lack of specific tools to measure their quality, and it remains unknown whether traditional ethnic dietary patterns can inform country-specific healthy diets, comparable to the Mediterranean and other evidence-based diets [29]. The Healthy Eating Index (HEI) measures dietary quality based on the Dietary Guidelines for Americans [30, 31]. Using HEI, the influence of dietary quality and composition on the risk of obesity and impaired blood glucose regulation can be assessed. We therefore investigated the relationship of HEI with glucose profiles and *Bifidobacterium* counts in two ethnic groups of Indonesian women with diverse diets.

## Materials and methods

### Subjects and study design

A cross-sectional study was conducted between September to November 2016 in districts representing mountainous and coastal areas (Tanah Datar and Padang Pariaman districts in West Sumatera province for the Minangkabau ethnic group, and Tasikmalaya district in West Java province for the Sundanese ethnic group). People in mountainous areas more frequently consume salted fish or fresh fish, if they have their own fish pond, while people in coastal areas consume predominantly fresh fish up to six times a week [32]. Both areas have agricultural fields, and the population of farmers and fishermen is above the regional average. Specific villages and hamlets in this study were randomly selected by multi-stage random cluster sampling.

Subjects were those who met the following criteria: apparently healthy reproductive women aged 19–50 years old having both parents from the same ethnicities (Minangkabau or Sundanese), not being pregnant or lactating, not having symptoms of gastrointestinal disturbance in the last 2 weeks such as diarrhea, dysentery, constipation for more than 3 days and/or abdominal pain [33], not having nausea or vomiting or loss of appetite for the last 2 days, no history of malignancy, not consuming antibiotics in the last 1 week before fecal collection [7], and not consuming alcohol more than 3 times a week [34]. Before the start of the study, all subjects who were willing to participate voluntarily signed a written informed-consent. The study was approved by both the Research Ethics Committee, Faculty of Medicine, Universitas Indonesia and Dr. Cipto Mangunkusumo General Hospital, as well as the Directorate General of National Unity and Politics, and the public health office in both provinces. This study is registered at clinicaltrial.gov, number NCT03412617.

A minimum sample size of 238 women (119 from each ethnic group) was required to detect an association between HEI and total *Bifidobacterium* with multiple linear regression analysis [11, 35] (α = 5%, β = 20%, R^2^ = 0.2, independent variable = 5) assuming an estimated non-response rate of 10%, and multiplied by 1.9 to accommodate the design effect of clustered sampling.

**Fig 1:**
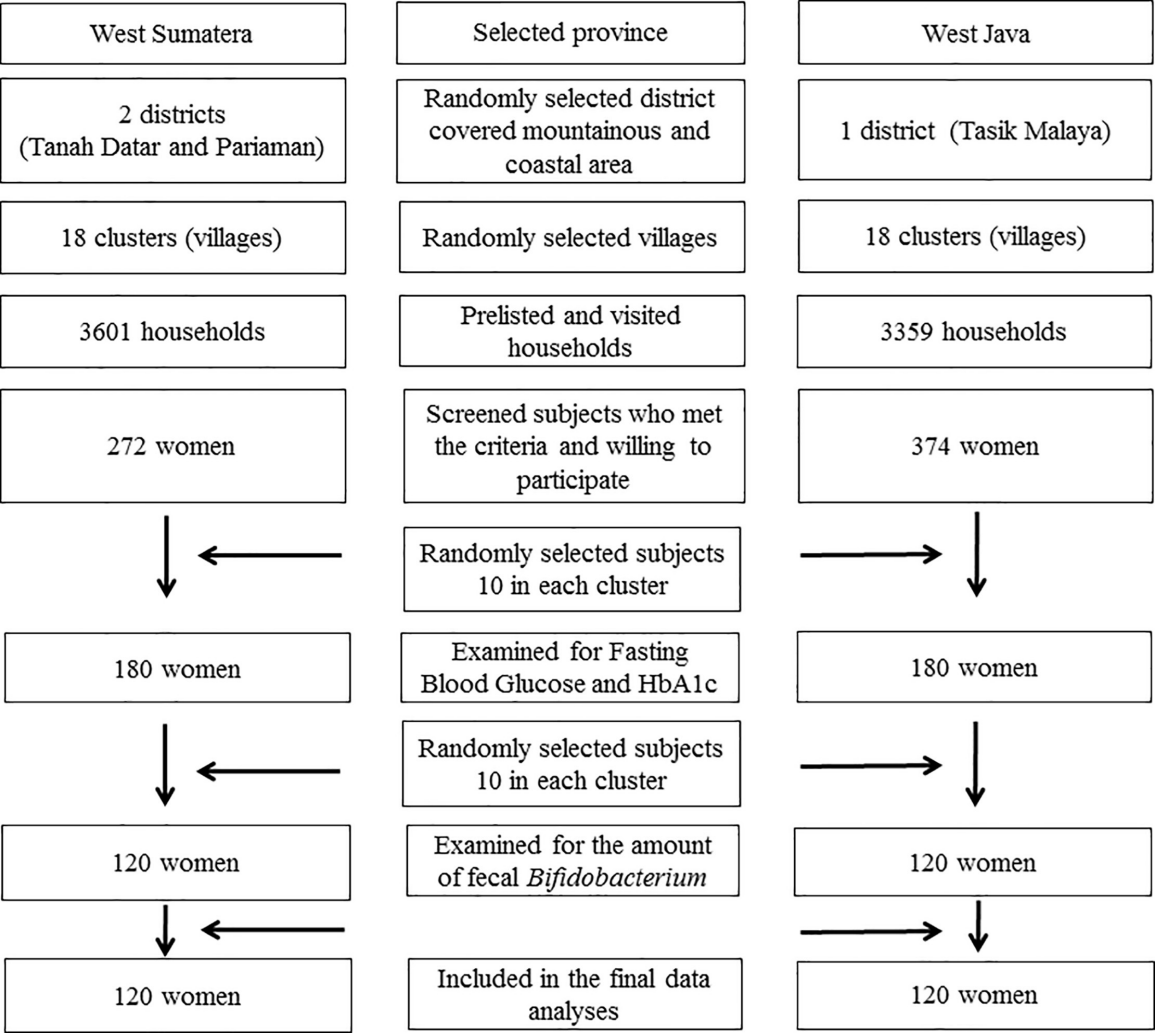
Flow of cluster sampling and subject recruitment.

Subjects were selected by using probability proportional to population size as can be seen in **Fig 1**. First, 18 villages in each province (total 36 villages) were randomly selected using the Emergency Nutrition Assessment software (ENA 2011). From each village, one cluster consisting of a maximum of 200 households was randomly selected, such that 18 clusters were formed per province. All women from the 200 selected households were included who met the criteria. From those, we randomly selected 10 from each cluster. If the required number of women was not acquired, the closest cluster was included to obtain the required number of subjects. The total subjects in this study were 360 women, while *Bifidobacterium* was examined in a randomly selected subgroup of 120 subjects from each district (n = 240).

### Data collection

Field enumerators were recruited based on their academic performance, high motivation, at least a bachelor’s degree or diploma and majoring in nutrition or public health, experienced in field research, especially on anthropometric measurement and interviewing to assess food intake. They were trained to carry out standardized dietary recalls and for proper stool sampling procedures.

Before data collection, surveys of traditional markets were done to determine the availability and prices of foodstuffs, and focus group discussions were conducted involving 10 women, and 3 household visits to observe cooking processes in each province to better interpret reported dietary intake, and to adjust for other field conditions during the study.

### Dietary assessment

Dietary intake was assessed using 2 day-repeated 24-hour food recalls on non-consecutive days that included weekdays and weekends. The HEI 2010 was used to assess the quality of nutrient intake and food groups, which consist of 12 components; nine for the adequacy for healthy foods (the greater the intake, the higher the score) and three components for moderation (the lower the intake, the higher the score obtained). A higher total score indicates intakes in greater accordance with dietary guidelines. The HEI 2010 has been calibrated and validated by the United States Department of Agriculture (USDA) for all American demographic groups, including children, adults, the elderly, and pregnant and lactating women. The HEI 2010 has not been specifically adapted for certain ethnic or cultural groups, but can be assumed to be applicable in populations where diets can be classified into the existing components of the tool [30]. Although the HEI has not been validated in Indonesia, there are many countries in Asia that obtained valid findings using the HEI, for example Malaysia [36], Thailand [37]; and other middle and low income countries [38].

Indonesian dietary guidelines (IDG) closely resemble the Dietary Guidelines for Americans (**Table 1**): a serving portion of one meal is composed of staple foods (rice, wheat, bread, sago, corn, tubers, etc.), vegetables, fruits, and protein sources [39, 40]. The amounts of fruit and food protein sources should be less than staple foods and vegetables. In the IDG, consumption of whole grains was not differentiated from refined grains. As such, scoring of the whole grains component in the Indonesian population would be underestimated. The suggested portion of milk consumption is not included in IDG, and would result in a lower HEI, whereas US dietary guidelines clearly mention 3 portions (glass) of milk a day. Also, the score for empty calories may be overestimated because alcohol consumption in Indonesian women is 0.1 L per capita per year, much lower than for American women at 4.9 L [41]. The specific components of the HEI can be seen in more detail elsewhere [30]. For HEI scoring of saturated, monounsaturated, and polyunsaturated fatty acids we used food composition tables of Thailand, Vietnam, and the United States. Analysis of dietary intake was performed using Nutrisurvey 2007 software [42].

**Table 1:** Differences between Indonesian and American dietary guidelines.

Indonesian		American		Differences
Component	Total Consumption/day	Component	Total Consumption/day	
**Calories**	2150–2725	Calories	2200–2800	-
**Grains, roots, and tubers**	3–4 portions	Whole grains Refined grains	3.5–5 portions 3.5–5 portions	INA DG does not define whole and refined grains
**Vegetables**	3–4 portions (21–28 portions /week)	Vegetables Legumes	19–22.5 portions/week 2–2.5 portions	INA DG does not separate vegetables and legumes
**Fruits**	2–3 portions	Fruits	2–2.5 portions	-
**Plant Protein (30%)**	2–3 portions	Nuts, seeds, soy products	5 portions/week	INA DG does not emphasize consumption of dairy products at every meal
**Animal Protein (70%)**	2–3 portions (21 portions/week)	Meat and seafood Dairy	37–43 portions/week 3 portions	
**Oils**	5–7 portions (25–35 g)	Oils	29–36 g	-
**Other calories**	350 kcal (12%)– 400 kcal (14.5%)	Other calories	280 kcal (13%)– 400 kcal (14%)	Alcohol consumption in Indonesian people is much lower than American

Note: INA DG, Indonesian Dietary Guidelines

Edited from references [39] and [40]

### Anthropometric measurement

Anthropometry was carried out using a SECA scale type 876 for weight to the nearest 0.1 kg, and a 2m Shorr Board for height to the nearest 0.1 cm by professionals who had passed our training course. All participants were required to wear only light clothing and stand erect, barefoot, and at ease while being measured [43]. Both weight and height measurements were performed twice, and the average was used to calculate body mass index (BMI). BMI classifications were based on the Asia Pacific classification system [44]. Asian people, especially South Asians, are more likely to develop metabolic disease at a lower BMI because they have less muscle and more abdominal fat, which increases insulin resistance [45]. According to the Asia Pacific Guidelines, underweight was defined as BMI <18.5 kg/m^2^, normal was ≥18.5 and <23 kg/m2, overweight was ≥23 and <25 kg/m^2^, obesity was ≥25 kg/m^2^ [46].

### Laboratory examination

Sampling and analysis of venous blood and *Bifidobacterium* were done in collaboration with commercial laboratories routinely performing the assays for clinical purposes. Subjects were required to fast at least 12 hours up to a maximum 14 hours before collection of 10 ml venous blood from the cubital fossa into vacutainers containing EDTA. Fasting blood glucose (FBG) was quantified using the enzymatic colorimetric method with glucose oxidase–phenol aminophenazone, and HbA1c was done using high performance liquid chromatography (HPLC) following hexokinase treatment.

Fecal samples were collected in 2 pots, each containing 5–10 gram of stool, and stored in a cool box (2–9°C) until being transported to a laboratory and stored in a -80°C freezer. DNA from the fecal sample was extracted using TianAmp Stool DNA Kit (DP 328). The concentration of total DNA was determined with a Nanodrop Termo BMS ND 2000 spectrophotometer, and quantification of *Bifidobacterium* DNA was done using the *Bifidobacterium sp*. (CGGGTGAGTAATGCGTGACC) standard primer and using real-time quantitative Polymerase Chain Reaction (Applied Biosystem (ABI) 7500 Real Time PCR System) [47].

### Internal validity

Collection of dietary intake data and determination of the HEI score were validated for all ethnic food items. We trained and standardized enumerators in the 24-hour food recall method both before and during data collection, and we used a food consumption photograph book with images of local foods to help enumerators and subjects determine the type and portion size of food. Food group categorization and HEI scoring was re-checked independently by two persons. Combined food dishes were separated into specific ingredients that were encoded separately. Physical activity level was assessed by the international physical activity questionnaire (IPAQ) short form. Blood and fecal sampling were done in accordance with operational standards, and the assessment of FBG, HbA1c, and *Bifidobacterium* count were examined in a standardized laboratory, and validated against controls.

### Statistical analysis

Data analysis was performed using SPSS version 20.0 [42]. Data were analyzed descriptively for plausible values using range checks and the Kolmogorov-Smirnoff normality test. HEI differences between Minangkabau and Sundanese women were analyzed using unpaired t-tests if the data were normally distributed or Mann-Whitney test for non-normal data. The association between HEI with FBG, HbA1c, and intestinal *Bifidobacterium* count was analyzed by multiple linear regression with a significance level of p <0.05. To identify potential confounders to include in the analysis, e.g. ethnic group, age, BMI, education level, income, physical activity, haemoglobin level, carbohydrate, protein, and fiber intake, univariate regression was used with a significance limit of p <0.25.

## Results

### Subjects

The total subjects examined for fecal *Bifidobacterium* was 120 from each district (n = 240), selected from 360 women involved in this study, as shown in **Fig 1**. Their characteristics are summarized in **Table 2**. The mean age of the Minangkabau women tended to be older than for Sundanese subjects. Minangkabau subjects had a higher education level and higher income compared to Sundanese subjects. There was a substantial difference in BMI, with about 73% Sundanese subjects being overweight or obese, compared to 57% for Minangkabau subjects (p = 0.014).

**Table 2:** Sociodemographic characteristics of Minangkabau and Sundanese women^1^.

Variable	Minangkabau (n = 120)	Sundanese (n = 120)	All (n = 240)	p
**Age (year)**	40.0 (31.0–45.0)	37.0 (30.3–42.8)	38.0 (31.0–44.0)	0.073
**Education level**				
**< 9 years**	22 (18.3)	61 (50.8)	83 (34.6)	0.000*
**≥ 9 years**	98 (81.7)	59 (49.2)	157 (65.4)	
**Household income (IDR 1.000K)** ^**2**^	1.5k (1–2)k	1.0k (0.8–2)k	1.46k (0.9–2)k	0.037*
**Classification**				
**Low**	79 (65.8)	68 (56.7)	147 (61.3)	0.145
**Sufficient**	41 (34.2)	52 (43.3)	93 (38.8)	

^1^ Variable presented in mean ± SD; median (25th percentile-75th percentile); or n (%)

^2^ Provincial minimum wage of West Sumatera = IDR 1,800,725 (USD 133); Provincial minimum wage of West Java = IDR 1,300,000 (USD 96)

* statistically significant (p<0.05)

### Healthy Eating Index, glucose profile, and total Bifidobacterium count

Most of the subjects had HEI scores <51, as shown in **Table 3**, suggesting they had poor dietary quality. The mean HEI of Minangkabau subjects was 33.6 ± 8.0 and tended to be slightly higher than Sundanese 32.1 ± 6.7. No subject had an HEI score higher than 80, and only three Minangkabau women and one Sundanese woman had HEI scores between 51 and 80, underscoring the need for improved dietary quality.

**Table 3:** Healthy Eating Index score of Minangkabau and Sundanese women^1^.

Component	Max Score	Minangkabau (n = 120)	Sundanese (n = 120)	All (n = 240)	p
**Total HEI**	100	33.6 ± 8.0	32.1 ± 6.7	32.9 ± 7.4	0.096
**Component HEI**					
**Total fruit**	5	0.96 (0.18–2.5)	0.44 (0–2.0)	0.74 (0–2.4)	0.005*
**Whole fruit**	5	1.9 (0.4–2.8)	0.88 (0–2.5)	1.5 (0–2.5)	0.014*
**Total vegetable**	5	2.3 (1.6–3.5)	2.2 (1.2–3.2)	2.2 (1.4–3.3)	0.083
**Greens & beans**	5	0.76 (0–2.5)	0.59 (0–2.3)	0.65 (0–2.5)	0.640
**Whole grains**	10	0 (0–0)	0 (0–0)	0 (0–0)	1
**Dairy**	10	0 (0–0)	0 (0–0.01)	0 (0–0)	0.222
**Total protein foods**	5	4.8 (3.8–5.0)	3.7 (2.8–4.9)	4.4 (3.2–5.0)	<0.001
**Seafood & plant protein**	5	4.5 (2.9–5.0)	3.5 (2.5–4.9)	3.9 (2.6–5.0)	0.002*
**Fatty acids**	10	0 (0–0)	0 (0–0)	0 (0–0)	0.157
**Refined grains**	10	0 (0–0)	0 (0–0)	0 (0–0)	0.005*
**Sodium**	10	6.7 (5.0–9.2)	0.79 (0–4.8)	4.9 (0.24–7.6)	<0.001*
**Empty calories**	20	11.8 (7.3–16.3)	17.9 (14.3–20.0)	15.4 (10.0–19.3)	<0.001*

HEI: Healthy Eating Index

^1^ Variable presented as mean ± SD or median (25th percentile-75th percentile)

* Statistically significant (p<0.05)

The median HbA1c and mean of intestinal *Bifidobacterium* in Minangkabau women were significantly higher than for Sundanese (**Table 4**). Before multiple linear regression analysis to assess the relationship of HEI to FBG, HbA1c, and intestinal *Bifidobacterium*, univariate regression was conducted to determine potential confounders. Ethnic group, age, BMI, education level, income, physical activity, hemoglobin level, and carbohydrate, protein, and fiber intake emerged as significant confounders. We further note that for *Bifidobacterium* levels the significant confounders were ethnic group, BMI, education level, and income; while for FBG we identified age and BMI as confounders; and for HbA1c we identified ethnic group, age, and BMI.

**Table 4:** Body mass index, fasting blood glucose, HbA1c, and intestinal *Bifidobacterium* of Minangkabau and Sundanese women^1^.

Variable	Minangkabau (n = 120)	Sundanese (n = 120)	All (n = 240)	p
**BMI (kg/m**^**2**^**)** ^**2**^	24.2 ± 4.6	25.5 ± 4.2	24.9 ± 49.5	0.025*
**Classification**				
**Underweight**	12 (10.0)	2 (1.7)	14 (5.8)	
**Normal**	39 (32.5)	30 (25.0)	69 (28.8)	0.014*
**Overweight**	17 (14.2)	21 (17.5)	38 (15.8)	
**Obese**	52 (43.3)	67 (55.8)	119 (49.6)	
**FBG (mg/dL)**	76.0 (70.3–81.0)	77.0 (71.0–84.0)	77.0 (71.0–83.0)	0.269
**HbA1c (%)**	5.5 (5.2–5.9)	5.4 (5.2–5.6)	5.4 (5.2–5.7)	0.022*
**Intestinal *Bifidobacterium* (log cell/g feces)**	8.98 ± 0.69	8.73 ± 0.67	8.9 ± 0.69	0.004*

FBG, Fasting Blood Glucose

^1^ Variable presented in mean ± SD or median (25th percentile-75th percentile)

^2^ Underweight = BMI<18,5 kg/m^2^; Normal = BMI 18,5–22,9 kg/m^2^; Overweight = BMI 23,0–24,9 kg/m^2^; Obese = BMI ≥25,0 kg/m^2^

*statistically significant (p<0.05)

**Fig 2:**
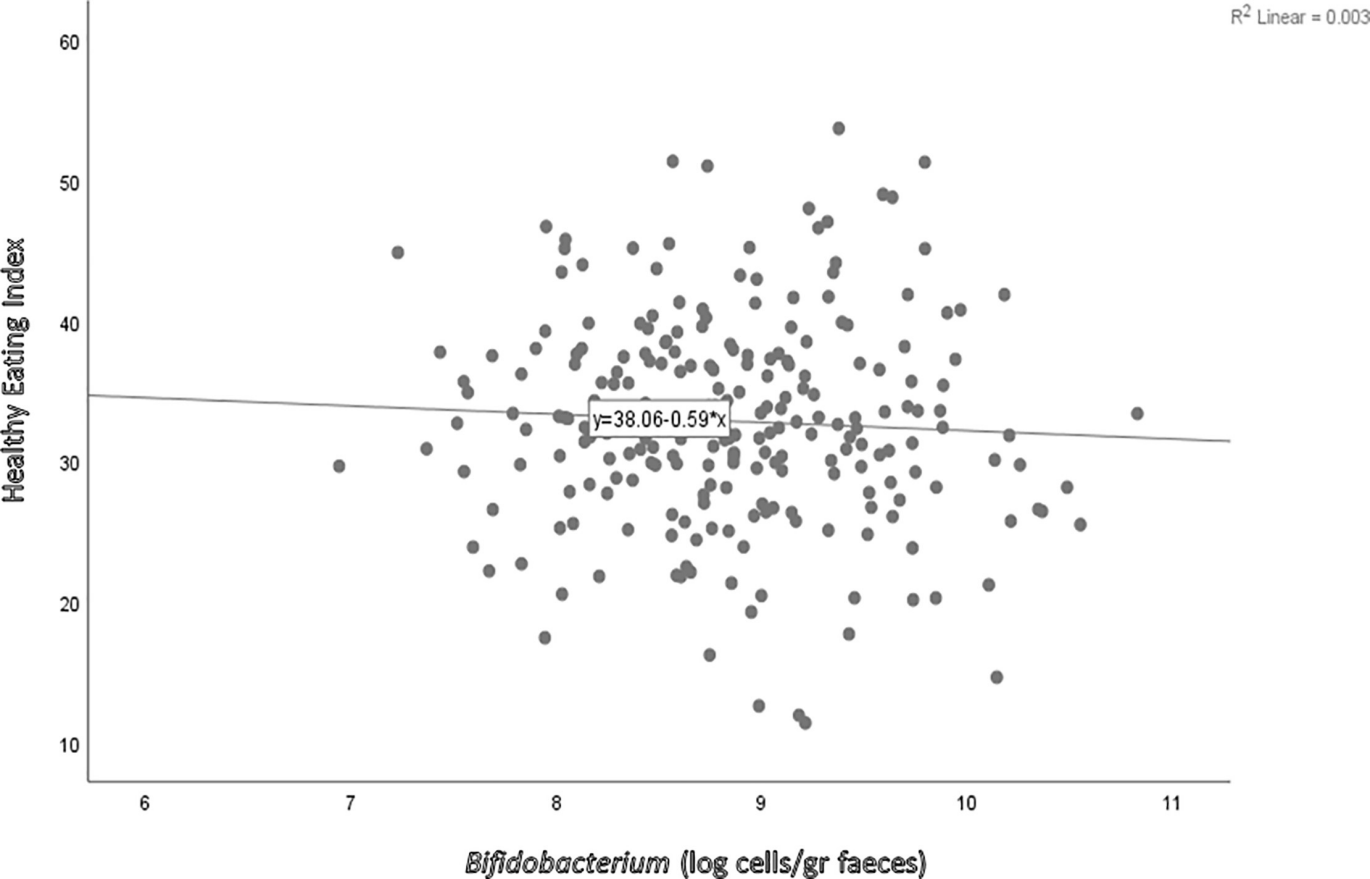
Scatterplots of Healthy Eating Index 2010 versus fecal *Bifidobacterium* counts among women in West Sumatera and West Java Provinces.

Results from multiple linear regression show an association between HEI with FBG and HbA1c, but no association between HEI with the number of *Bifidobacterium* in Minangkabau or Sundanese women as shown in **Fig 2** and **Table 5**. BMI significantly predicted FBG (*ß* = 0.004; 95% CI = 0.306–1.588; p = 0.004) and HbA1c (*ß* = 0.053; 95% CI = 0.022–0.083; p = 0.001), while ethnic group was associated with *Bifidobacterium* (*ß* = -0.213; 95% CI = -0.398–0.029; p = 0.024).

**Table 5:** Relationship of Healthy Eating Index with fasting blood glucose, HbA1c, and intestinal *Bifidobacterium* counts in Indonesian women by linear regression analysis (n = 240).

Variable	Unadjusted β	95% CI	p	Adjusted β	95% CI	P
	**Fasting Blood Glucose**					
HEI	-0.436	-0.828- (-0.043)	0.030*	-0.403	-0.789- (-0.016)	0.041*
Age				0.165	-0.206–0.536	0.382
BMI				0.004	0.306–1.588	0.004*
	**HbA1c**					
HEI	-0.020	-0.039-(-0.001)	0.043*	-0.018	-0.036–0.000	0.048*
Tribe				-0.198	-0.472–0.075	0.155
Age				0.015	-0.001–0.034	0.061
BMI				0.053	0.022–0.083	0.001*
Hemoglobin				0.127	0.048–0.206	0.002
		**Intestinal *Bifidobacterium* Count**				
HEI	-0.005	-0.017–0.007	0.400	-0.007	-0.018–0.005	0.275
Tribe				-0.213	-0.398–0.029	0.024*
BMI				-0.014	-0.034–0.006	0.167
Education level				0.074	-0.124–0.272	0.462
Income				0.032	-0.040–0.104	0.380

CI: *Confidence Intervals*; HEI: Healthy Eating Index

* statistically significant (p <0.05)

Linear regression analysis of the association between *Bifidobacterium* counts with FBG and HbA1c in Minangkabau and Sundanese women showed no relationship after adjusting for multiple confounders as shown in **Table 6.**

**Table 6:** Relationship of intestinal *Bifidobacterium* count to fasting blood glucose and HbA1c in Minangkabau and Sundanese women using linear regression analysis (n = 240).

Variable	Unadjusted β	95% CI	p	Adjusted β	95% CI	p
	**Fasting Blood Glucose**					
*Bifidobacterium*	1.452	-2.811–5.715	0.503	2.341	-1.889–6.571	0.277
Age				0.173	-0.202–0.547	0.364
BMI				1.024	0.374–1.674	0.002*
Fiber intake				-0.092	-0.695–0.511	0.764
	**HbA1c**					
*Bifidobacterium*	0.065	-0.142–0.272	0.537	0.093	-0.107–0.294	0.360
Tribe				-0.133	-0.416–0.150	0.354
Age				0.017	0.000–0.035	0.052
BMI				0.055	0.023–0.086	0.001*
Hemoglobin				0.131	0.052–0.211	0.001*
Fiber intake				-0.008	-0.107–0.294	0.568

CI: *Confidence Interval*

* statistically significant (p <0.05)

## Discussion

Dietary quality of women scored by HEI 2010 is clearly associated with measures of blood glucose, FBG and HbA1c, after adjustment for confounders. An increase of one point in HEI score decreased FBG by 0.403 mg/dL and decreased HbA1c levels by 0.18‰. However, this study did not observe a relationship between HEI and intestinal *Bifidobacterium* counts after adjusment for ethnic groups (Minangkabau and Sundanese), BMI, education level, and income. The mediating role of *Bifidobacterium* between dietary quality and glucose outcomes was not apparent as *Bifidobacterium* count was not significantly associated with levels of either FBG or HbA1c. A previous study by Allin et al. showed that individuals with prediabetes had aberrant intestinal microbiota characterised by a decreased abundance of the genus *Clostridium* and the mucin-degrading bacterium *A*. *muciniphila* [48].

The mean of HEI score in the Minangkabau women (33.6 ± 8.0) tended to be higher than for Sundanese women (32.1 ± 6.7). The overall values indicated poor diets, i.e. scores less than 51 [49, 50]. However, it is difficult to compare the current findings with other studies in Indonesia because others did not use the HEI score in adult women. One study in Indonesia that used a one-day 24-hour food recall was the 2010 National Health Research Survey wherein the mean score of adult women was 31.0 ± 12.1 out of a possible maximal score of 100 [26]. This is similar to the HEI score in our study. We note the average score of HEI in adults was 66 in Macau [51], 68 in Brazil [52] and 63 in the United States (72% of scores between 51–80 and 18% of scores ≤50, n = 10,930) [53], which are much higher than our study population.

Low HEI scores in women may be due to low overall food intake or low diversity of food consumption. Low food intake can be caused by the consumption of only certain [39] or favorite foods, and low socioeconomic conditions [26]; and low diversity may be influenced by lack of knowledge and awareness of dietary habits and quality, composition of the household, food availability and ecological factors, food purchasing power, and time available for food processing [54, 55]. In this study, all subjects lived in rural areas and most were housewives who may often stay at home and eat the same types of foods on a daily basis. The low HEI scores in the present study may be due to low education and income level of women and their families. Due to limited funds, they often consume white rice with one kind of side dish, or food snacks that are more affordable, rather than purchasing all the ingredients for cooking. In this study, the median HEI score of zero can be explained as follows: all subjects ate white rice as a primary carbohydrate source, and consumed other grains from processed snacks and fast food with refined grains, in contrast to traditional snacks such as lamang (glutinous rice cooked with coconut milk), kue beras and salalauak (West Sumatera’s traditional food made from rice flour), and with low intake of dairy products regarded as luxurious and expensive foods, and with high consumption of fried foods and coconut milk from traditional foods. The differences between Indonesian and USDA dietary guidelines may also affect HEI scores, for example Indonesian dietary guidelines do not emphasize consumption of whole grains, nor to drink milk three times a day. As mentioned, although formal validation of the HEI has not been done in Indonesia, the HEI has been used for research in Asian countries with useful and interpretable findings [36, 37].

The association of dietary quality with FBG is consistent with a study that used an alternate HEI scoring process and showed negative correlations between dietary quality and changes in HbA1c [56]. Alison et al. indicated that patients with type 2 diabetes mellitus had lower HEI scores [57]. The significant association between HEI with FBG and HbA1c in Minangkabau and Sundanese women in this study may be due to insulin sensitivity that could be modulated by multiple environmental factors, especially dietary habits. The influence of diet on insulin sensitivity is mediated by its energy content and nutrient composition, in particular by different types of dietary fatty acids [58]. In Sundanese women, fasting blood glucose tended to be higher, but the HbA1c was significantly lower than in Minangkabau women. This result shows that FBG may not always yield the same result as HbA1c because FBG shows the current blood glucose levels, while HbA1c reflects the blood glucose levels over the last 3 months.

The average number of *Bifidobacterium* in the Minangkabau group was 8.98 ± 0.69 log cells/gram of feces and was significantly higher than the Sundanese at 8.73 ± 0.67 log cells/gram of feces. This is consistent with previous studies suggesting that ethnic and geographic differences may affect the composition of the intestinal microbiota [59]. An ideal microbiota balance is required and gut microbiota are critical maintain intestinal epithelial barrier function and physiological homeostasis.[60] The number of *Bifidobacterium* in our subjects was not much different from the results of previous studies with the average number of 8.6 ± 1.2 log cells/gram of feces in healthy adults [61]. The results of a study of 46 Japanese healthy adults showed that the average *Bifidobacterium* count was higher (9.4 ± 0.7 log cells/gram of feces) than our findings [62]. However, the amount of intestinal *Bifidobacterium* in adult women in general, or various ethnicities in Indonesia, was previously unknown.

Differences in the number of microbiota and its composition in various ethnic groups and different regions have been reported [59, 63–65]. High numbers of *Bifidobacterium* and *Bacteroides* species were found in six cities of China, Japan, and Taiwan that consumed higher levels of animal protein, compared to those in Indonesia and Thailand which consume more carbohydrates, and had higher levels of *Prevotella* species [66]. Cultural differences and residential location will also affect the type of food, the availability of foodstuffs, as well as many other factors that may affect microbiota [67], and vertical transmission of microbiota and microbiota genes. Diet is a major factor and contributes as much as 57% of the formation of intestinal microbiota composition, while the genetic effect is 12% [8]. Food patterns (long-term) and food variations (short-term) affect the composition of the intestinal microbiota, including the amount of *Bifidobacterium* [68].

No previous studies have examined the relationship between HEI and intestinal *Bifidobacterium* count. Several studies showed that HEI was associated with various health markers, was a significant predictor of BMI and waist circumference in multi-ethnic populations [69], had a positive correlation with diversity of food and total energy, micronutrient and fruit intake, and had a negative correlation with fat and saturated fatty acids intake [31, 70]. The high intake of fat and saturated fats can lead to a decrease in the amount of intestinal *Bifidobacterium* [71].

The lack of any relationship between HEI and *Bifidobacterium* counts in this study may be due to other factors that affect *Bifidobacterium*. For example, ethnic group had a significant effect on *Bifidobacterium* counts. In addition, besides fat and saturated fatty acids intake, HEI is strongly associated with fruit intake and food diversity [31]. Intake of carbohydrates, proteins, fiber, and PUFA also affect the composition of *Bifidobacterium* [7, 8, 15, 72]. From several studies, it was found that a high-fat diet was associated with a decrease in the number of gram-positive (*Bifidobacterium sp*.) and gram-negative (*Bacteroides*) bacteria, and also increased *Firmicutes* and *Proteobacteria* [18, 68, 71], but some studies showed otherwise [7]. Notably, the amount of *Bifidobacterium* is also influenced by overall bacterial composition, genetics and age.

Minangkabau subjects with a lower BMI had a higher *Bifidobacterium* level than the Sundanese. This is consistent with previous studies, which suggested that the composition of intestinal microbiota is related to body weight and obesity [73]. Significant associations in previous studies were increased counts of *Lactobacillus*, *Staphylococcus aureus*, *Escherichia coli*, and *Faecalibacterium prausnitzii* in obese subjects, and a decrease in *Bifidobacterium* counts [74–77]. A previous study by Gao et al. found that *Bifidobacterium* counts were significantly more abundant in healthy volunteers compared with the obese patients [78]. These multiple studies with disparate results suggest a complex relationship between microbiota and excess body weight, and knowledge of which microbiota influence obesity remains unknown [8, 79].

High fat and saturated fat intake can lead to dysbiosis of gut microbiota and reduce the number of *Bifidobacterium* [71] through several mechanisms i.e. decreased activity of intestinal alkaline phosphatase (IAP) [80–84], oxidative stress, and increased amounts of the bile acid deoxycholic acid in the intestine [85–87]. Fiber intake also affects the amount of *Bifidobacterium* depending on the type and amount. The fermentable fibers will increase the bacterial mass by keeping the intestinal pH low so that it inhibits the growth of pathogenic bacteria [88]. *Bifidobacterium* can inhibit exogenous cholesterol absorption from the small intestine and may inhibit body weight gain [89].

One limitation of our study is we examined only *Bifidobacterium sp*., but did not assess the overall composition of microbiota in feces. Nevertheless, the data serve as a reference for future studies of dietary intake, especially for indigenous food of various ethnic groups in Indonesia and their relationship to gut microbiota and metabolic disease. We also note that education about dietary intake and food choice are needed because both Minangkabau and Sundanese women had low HEI scores and high saturated fatty acid intake.

In conclusion, this study shows that for Minangkabau and Sundanese Indonesian women consuming traditional ethnic diets, a lower HEI increased the risk of elevated FBG and HbA1c, but the mediating role of intestinal *Bifidobacterium* was not apparent. It is necessary to explore healthier traditional ethnic diets to better understand their impact on health. Although the links herein with gut microbiota were not apparent, analysis of microbiota using DNA sequencing methods would be useful to know the number and proportion of each group of bacteria that might be associated with diet and health.

## Supporting information

S1 File DataShare. (XLSX)
