# Correction: Dietary quality of predominantly traditional diets is associated with blood glucose profiles, but not with total fecal *Bifidobacterium* in Indonesian women

**DOI:** 10.1371/journal.pone.0215533

**Published:** 2019-04-18

**Authors:** The PLOS ONE Staff

## Notice of republication

An incorrect version of S1 File was published in error. The publisher apologizes for this error. This article was republished on April 1, 2019 to correct for this error. Please download this article again to view the correct version.
